# A 8-year retrospective clinical analysis of 272 patients of epidemic Keratoconjunctivitis in Beijing, China

**DOI:** 10.1186/s12886-019-1266-z

**Published:** 2019-12-19

**Authors:** Wenbo Hou, Xuguang Sun, Jun Feng, Yang Zhang, Zhiqun Wang

**Affiliations:** 1 Eye Hospital, China Academy Of Chinese Medical Sciences, Beijing, China; 2 Department Of Ocular Microbiology, Beijing Institute Of Ophthalmology, Beijing Tongren Hospital, Capital Medical University, Beijing, China

**Keywords:** Epidemic keratoconjunctivitis, Multiple subepithelial corneal infiltrates

## Abstract

**Background:**

Epidemic keratoconjunctivitis (EKC) is one of the most common eye infections worldwide. The analysis of clinical manifestations in different age groups help better know the disease. This study aims to provide more detailed analysis of 272 cases of EKC in 8 years, describe the differences of the clinical features among different age groups, and establish new clinical grading criteria.

**Methods:**

272 individuals were reviewed (2011–2019) in Beijing China. All the patients were classified into 3 grades according to the new grading criteria. The typical clinical signs of EKC and the photographs of the multiple subepithelial corneal infiltrates (MSI) were collected and analyzed. The number of 3 grades among and within different age groups were compared. The incidence of the typical signs among and within different age groups were compared. The proportion of each region of the cornea involved by MSI were compared.

**Results:**

No significant differences were detected among the 4 groups in terms of the number of mild, moderate and severe cases, no matter in all-patients analysis (*P* = 0.271) nor in acute-phase-patients analysis (*P* = 0.203). The proportion of the severe cases was the highest among all patients (*P* = 0.000). Among the incidence of the typical signs, corneal involvement was the most common accounting for 69.8% (*P*<0.05). The probability of central region involvement was significantly higher than that of pericentral region involvement (*P* = 0.015) and peripheral region involvement (*P* = 0.000).

**Conclusions:**

Appropriate attention should be paid on EKC, because of the considerable proportion of severe cases, the high incidence of corneal lesion, and the high incidence of central region involvement of MSI.

## Background

Epidemic keratoconjunctivitis (EKC) is a highly contagious infectious disease, which is caused by adenoviruses [1]. In the acute phase, EKC might be indicated by such signs as: edematous eyelids, palpebral conjunctival follicles, hyperemia, chemosis, pseudomembranes, subconjunctival hemorrhages, superficial punctate keratitis (SPK) and tender palpable preauricular lymph node. Multiple subepithelial corneal infiltrates (MSI) during the subacute and chronic phases may occour and may persist for months to years [2], which can result in permanent visual loss [3]. EKC may be further complicated by dry eye and symblepharon formation [4].

The clinical characteristics of outbreaks of HAdV-54 conjunctivitis in Japan were well analyzed by Uemura et al. [5] and Akiyoshi et al. [6]. However, there have been few studies describing the clinical features of sporadic cases in large sample. Different clinical presentations of the EKC outbreak in premature babies and their parents were analyzed by Koçluk et al. [7]. However, to the best of our knowledge, the study of clinical manifestations in different age groups (the children, the adolescents, the adults and the aged) have not been reported before. Aoki et al. described the clinical features of adenoviral conjunctivitis at the early stage of infection. In this study the severity of follicular conjunctivitis was classified into three grades: mild, moderate and severe [8]. However, there has been no clinical grading criterion so far that has an important guiding value for clinical diagnosis and treatment. This study focuses on the tissues stated above. This study provides more detailed clinical analysis of 272 cases of EKC, and establishes new clinical grading criteria.

## Methods

### Study population

272 patients (153 male and 119 female) diagnosed of EKC were reviewed retrospectively over 8 years (2011–2019) from the Department of Ophthalmology in Tongren Hospital (Beijing, CHN), which is the largest eye center in China. Due to the retrospective nature of the study, informed consent was waived. The study was performed in accordance with the Declaration of Helsinki and was approved by the local ethics committee.

The inclusion criteria were: 1) the patients were diagnosed clinically as having EKC according to the typical clinical signs during the acute phase, such as: eyelid swelling, palpebral conjunctival follicles, hyperemia, conjunctival edema, pseudomembranes, subconjunctival hemorrhages, SPK and tender palpable preauricular lymph node. 2) the patients were diagnosed of EKC during the subacute and chronic phases according to the typical clinical signs, such as: MSI.

The exclusion criteria were: 1) the patients whose information was incomplete. For example, the time of onset or the symptoms or the clinical signs were not recorded in their records. 2) the patients were complicated with other eye diseases including allergic conjunctivitis and other inflammatory eye diseases. 3) the patients had a history of contact lens use, a history of herpetic eye disease, a history of trauma, and a history of ocular surgery.

### General information

The age, the gender, the onset seasons, the course of the disease, the typical signs including conjunctival edema, subconjunctival hemorrhages, pseudomembranes, corneal involvement, anterior chamber reaction, eyelid swelling, preauricular lymphadenopathy were collected and analyzed. Patients with EKC were divided into four groups according to the age: the children (group1, aged 3–12 years), the adolescents (group2, aged 12–18 years), the adults (group3, aged 18–60 years) and the aged (group 4, aged > 60 years). In bilateral cases, the data of the more severe eye was used in the study. The situation of dry eye, symblepharon secondary to EKC during the subacute and chronic phases would also be summarized.

### Clinical grading

The severity of EKC was classified into three grades on the basis of the course of the disease and the corneal involvement as follows: in mild cases, the course of the disease was about one week without corneal involvement; in moderate cases, the course of the disease was about one to two weeks combined with the corneal epithelial lesions. The epithelial lesions often disappeared when the conjunctival inflammation alleviated; and in severe cases, the disease lasted for more than two weeks, the inflammation affected the corneal subepithelium. The corneal lesions still could not be improved even after the conjunctival inflammation subsided. The subepithelial corneal involvement lasted for months to years.

### Analysis of MSI

The photographs of the MSI were collected and analyzed. The cornea was divided into three regions as follows [9]: the central region (0 mm to 3.0 mm diameter), pericentral region (3.0 mm to 6.0 mm diameter) and peripheral region (6.0 mm to 9.0 mm diameter). The probability of each region involved by MSI were compared among the different regions.

### Statistical analysis

Data analysis of the study was performed using SPSS statistics 23 software (IBM Corp., Armonk, NY, USA). The incidence or the constituent ratio were assessed using Pearson chi-square tests and, if the number of cases in a subgroup analysis was too low, Fisher’s exact tests. The number of mild, moderate and severe cases among and within different age groups were compared. The clinical data of patients who went to see a doctor in acute phase were collected, and the percentage of the typical clinical signs including conjunctival edema, conjunctival hyperemia, pseudomembranes, corneal involvement, anterior chamber reaction, eyelid swelling, preauricular lymphadenopathy were compared among and within different age groups. The probability of each region involved by MSI were compared among the different regions. A value of *P*<0.05 was considered as statistically significant.

## Results

### Demographic features

A total of 272 patients including 153 male (56.3%) and 119 female (43.8%) diagnosed of EKC were reviewed retrospectively over 8 years from 2011 to 2019 (Table 1). The average age of the patients was 39.3 ± 14.6 years because a considerable proportion of cases (223 cases; 82.0%) were adults. 192 cases attended the eye clinic during the acute phase; 80 cases came to see the doctor during the subacute or chronic phase. 30.2% of patients were attacked in winter, 29.9% in summer, 22.4% in autumn, and 17.5% in spring. The incidence of EKC showed a significant difference when compared among the four seasons (*P* = 0.001, Pearson chi-square test). The incidence of EKC in spring is lower than in summer(*P* = 0.001, Pearson chi-square test). There was no significant difference when the incidence of EKC was compared between summer and autumn(*P* = 0.05, Pearson chi-square test). The incidence of EKC in autumn is lower than in winter(*P* = 0.001, Pearson chi-square test).

**Table 1:**
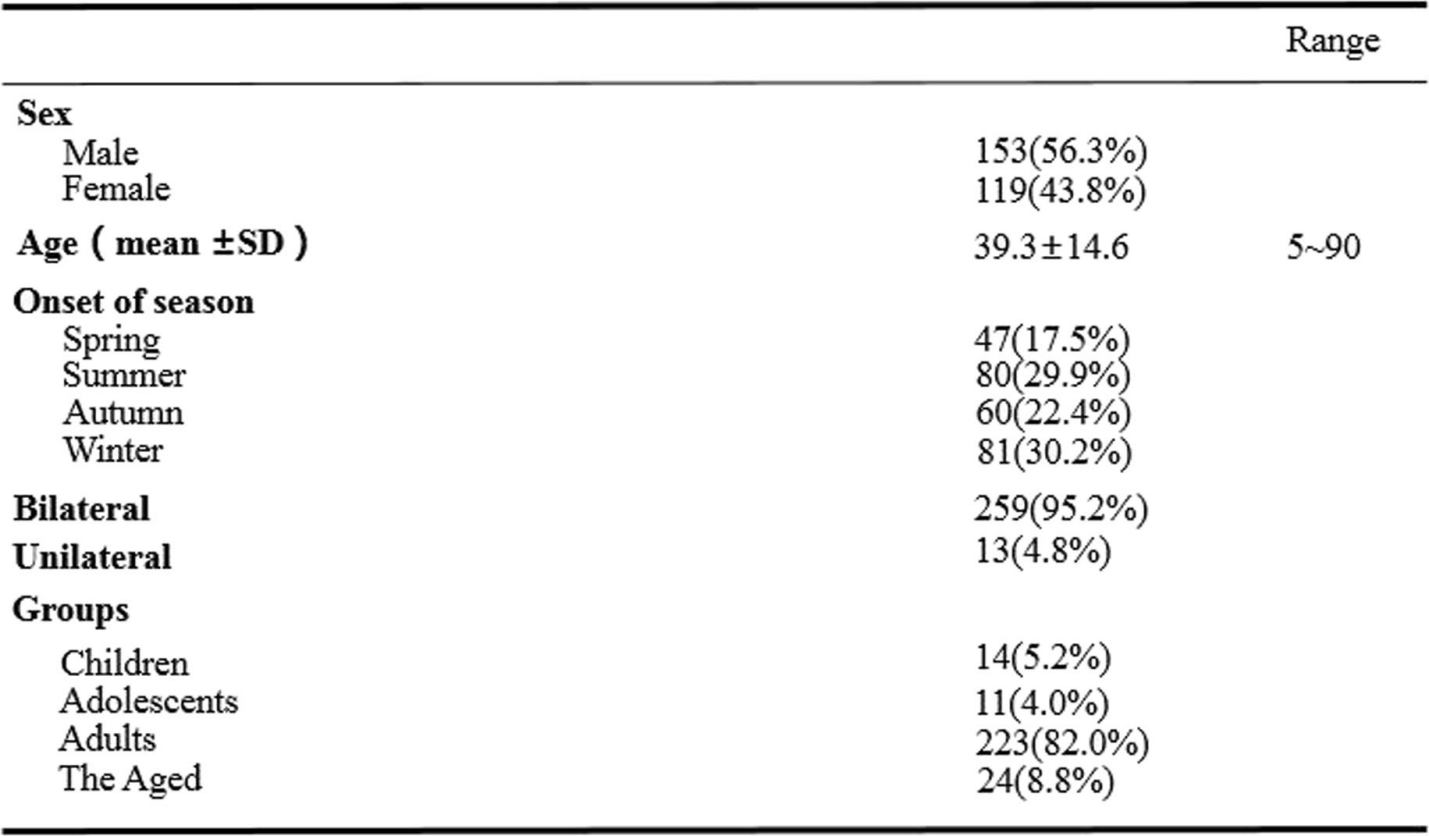
Demographic feature of 272 cases of epidemic keatoconjunctivitis

### Clinical grading

Table 2 demonstrates findings in relation to the clinical grading data. No significant differences were detected among the 4 groups in terms of the number of mild, moderate and severe cases (*P* = 0.271, Fisher’s exact test). Within the adults group (group 3), the number of severe cases showed a significant difference from those number of moderate cases (*P* = 0.000, Pearson chi-square test), and the number of moderate cases showed a significant difference from those number of mild cases (*P* = 0.000, Pearson chi-square test). Within the aged group (group 4), the number of severe cases showed a significant difference from those number of mild cases (*P* = 0.001, Fisher’s exact test). The severe cases accounted for the majority of the adults as well as the aged patients. It was found that the proportion of the severe cases was the highest among all patients (*P* = 0.000, Pearson chi-square test).

**Table 2:**
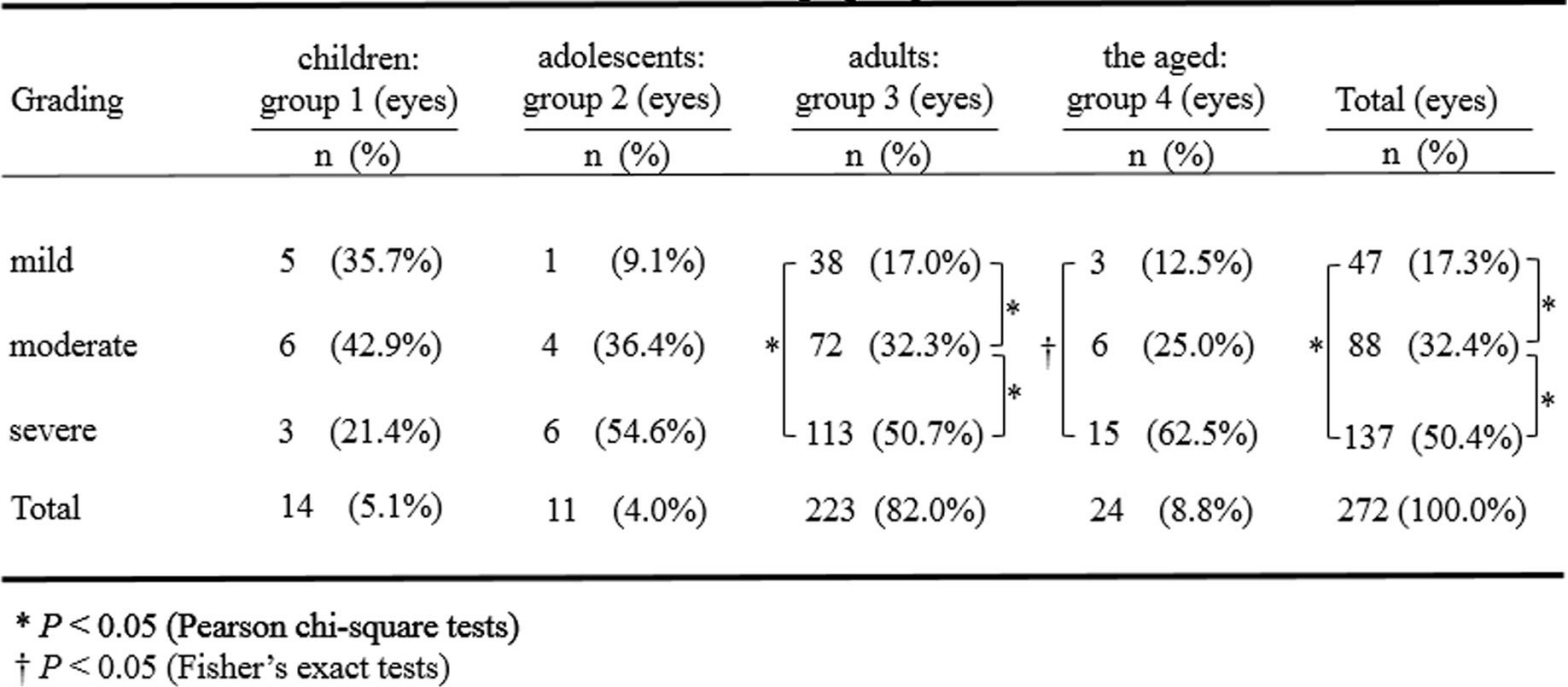
The differences in the clinical grading of epidemic keratoconjunctivitis among and within the differnt age groups

192 cases who attended the eye clinic during the acute phase were analyzed (Table 3). The number of moderate cases showed a significant difference from those number of mild cases (*P* = 0.000, Pearson chi-square test) and severe cases (*P* = 0.015, Pearson chi-square test). According to the analyses of the proportion of the mild, moderate, and the severe cases among the 4 groups, there was no significant difference (*P* = 0.203, Fisher’s exact test). The proportion of the moderate cases was significantly higher than that of mild cases (*P* = 0.000, Pearson chi-square test) and severe cases (*P* = 0.028, Pearson chi-square test) in the adults group (group 3). No cases showed severe grade in children group.

**Table 3:**
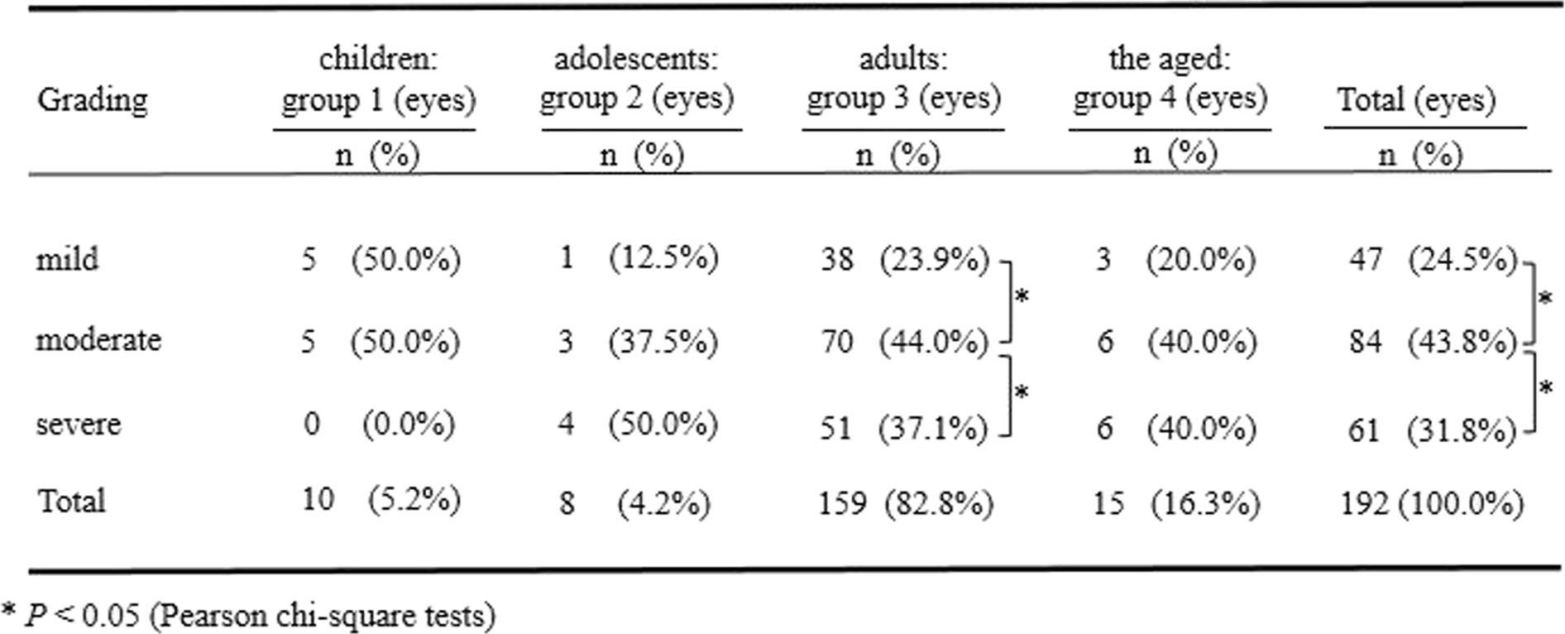
The differences in the clinical grading of epidemic keratoconjunctivitis among and within the different age groups during the acute phase

### Clinical signs

192 cases who attended the eye clinic during the acute phase were reviewed, the typical signs were analyzed (Table 4). Among the incidence of the typical signs, corneal involvement was the most common accounting for 69.8% (*P*<0.05, Pearson chi-square tests), followed by preauricular lymphadenopathy (29.2%), conjunctival edema (17.2%), subconjunctival hemorrhage (16.7%), pseudomembrane (15.1%), and eyelid swelling (15.1%). The incidence of the eyelid swelling is higher in the children group than in the adults group (*P* = 0.004, Fisher’s exact test) and the adolescents group (*P* = 0.013, Fisher’s exact test). In terms of the incidence of the eyelid swelling, there was significant difference among the 4 groups (*P* = 0.007, Fisher’s exact test). In the adults group, the incidence of corneal involvement was significantly higher than that of other signs (*P* = 0.000, Pearson chi-square test). Only one aged patient manifested keratic precipitates, and one child had anterior chamber reaction. One child, one adolescent and eight adults manifested filamentary keratitis during the subacute phase. 14.3% (39/272) of patients developed to dry eye. One child and one adult developed to symblepharon formation.

**Table 4:**
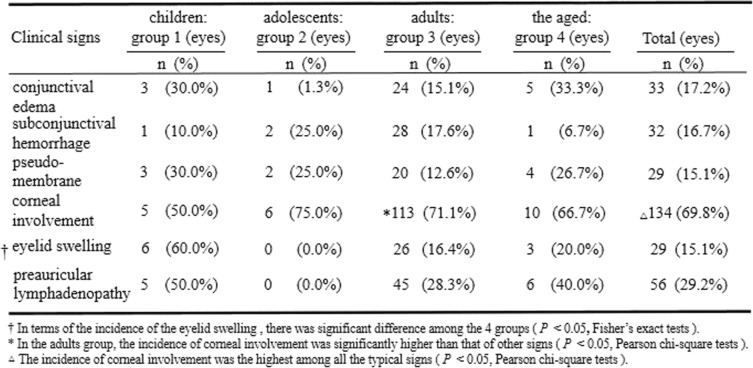
The differences in the clinical signs of epidemic keratoconjuctivitis among and within the different age groups during the acute phase

### Analysis of MSI

**Fig. 1:**
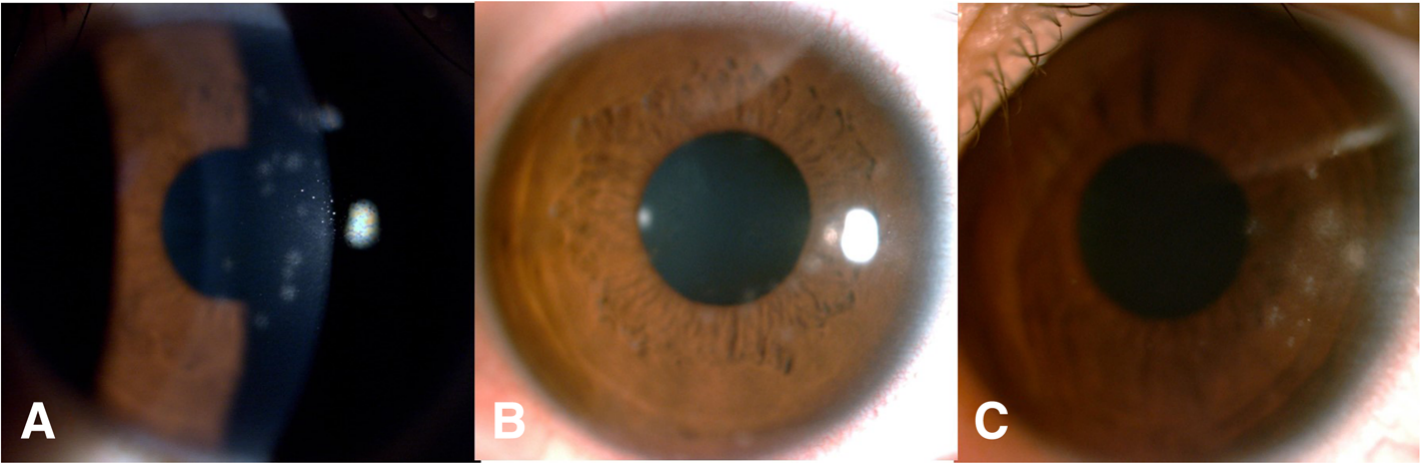
The distribution of the multiple subepithelial corneal infiltrates. **a**: The multiple subepithelial corneal infiltrates distributed in the central region of cornea. **b**: The multiple subepithelial corneal infiltrates distributed in the pericentral region. **c**: The multiple subepithelial corneal infiltrates distributed in the peripheral region

43 photographs of the MSI were collected and analyzed (Fig. 1). There was significant difference among 3 regions of cornea, in terms of the probability of each region involved by MSI (*P* = 0.000, Fisher’s exact test). The probability of central region involvement was significantly higher than that of pericentral region involvement (*P* = 0.015, Fisher’s exact test) and peripheral region involvement (*P* = 0.000, Fisher’s exact test) (Table 5).

**Table 5:**
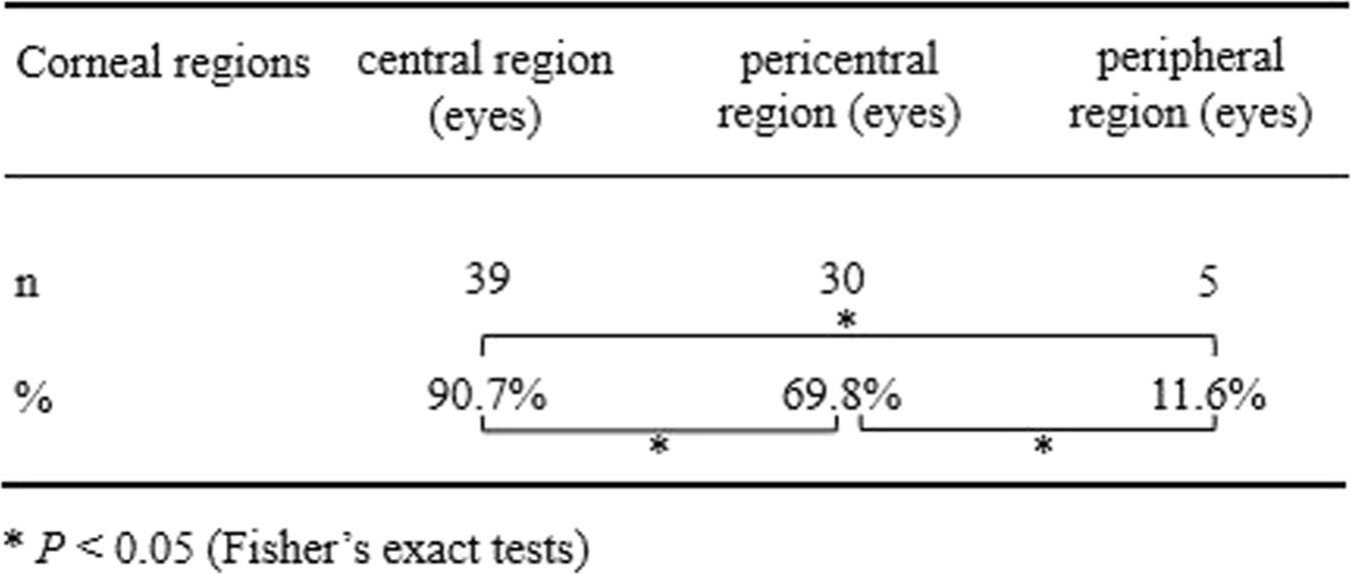
The differences of distribution of multiple subepithelial corneal infiltrates among the different corneal regions

## Discussion

EKC is one of the most common eye infections worldwide [10–13]. The clinical presentation of EKC is rather similar to the different adenovirus-type infections [11]. Most characteristic manifestation at the acute stage was the intense conjunctival hyperemia and edema, pseudomembranes, subconjunctival hemorrhages, superficial punctate keratitis (SPK) [14–17]. The involvement of the preauricular lymph node was found at this time [18–21]. After the acute phase, patients may develop multifocal, subepithelial, leukocytic infiltrates of the corneal stroma, which is called multiple subepithelial corneal infiltrates (MSI). These opacities may persist for months and even years with symptoms of visual impairment [22, 23].

EKC can cause large outbreaks worldwide reported by many researchers. For example, Muller et al. reported adenovirus-related EKC outbreak at a hospital-affiliated ophthalmology clinic [24]. Uemura et al. collected a consecutive series of 55 cases diagnosed clinically as EKC confirmed by HAdV-54 detection by polymerase chain reaction (PCR) method. The clinical findings were recorded and summarized [5]. Lei et al. reported the outbreaks of EKC caused by human adenovirus type 8 in the Tibet of China in 2016 [15]. However, EKC can also occur sporadically. There are considerable number of scattered cases in every season. Most of the patients attend the clinic without a clear history of infection or a contact history of red-eye patients. In our study, all 272 patients were sporadic cases over 8 years (2011–2019). To the best of our knowledge, there have been few studies describing the clinical features of sporadic cases in such large scale.

Akiyoshi et al. [6] and Koçluk et al. [7] described the clinical findings which were graded as 0, 1, 2, and 3. The scores of every sign were calculated and used for the statistics. However, this grading method makes a poor guide to the clinical work. There have been no clinical grading criteria so far that have an important guiding value for clinical diagnosis and treatment. In our study, the severity of EKC was classified into three grades according to the course of the disease and the corneal involvement. The grading criteria are useful because they can be performed easily and rapidly in clinical practice. Therapies can be differentiated from different grade.

According to the analysis of the 272 cases, there was no significant difference among the 4 different age groups, in terms of the proportion of mild, moderate and severe cases. According to the analyses of the 192 cases who attended the eye clinic in acute phase, there was also no significant difference among the 4 groups in terms of the proportion of mild, moderate, and severe cases. Which means that the proportion of the degree of EKC is the same in different age groups. According to the analysis of all the patients including acute and non-acute phase, the proportion of severe cases was the highest within the adults group, which is 50.7%. However, according to the analyses of the 192 cases in acute phase, the proportion of moderate cases was the highest within the adults group, which is 44.0%. Within the aged group, the severe cases contributed the largest proportion (62.5%) in the all-patient analysis, whereas the severe cases (40.0%) showed the same proportion as the moderate cases (40.0%) in the acute-phase-patient analysis. It was found that the severe cases accounted for was the highest among all patients. That means adults patients and aged patients should be paid appropriate attention, because of the considerable proportion of severe cases. They should be treated actively and timely. There was no severe case in children group, which may be related to the small amount of children patients to a certain extent. However, it does not mean that the children are less possible to turn to severer degree. Further study is needed in the future work.

The incidence of every typical sign during the acute phase was evaluated. The incidence of eyelid swelling is higher in the children group than that in the adults group and in the adolescents group. That means when a child manifested as eyelid swelling with acute-onset, efforts should be done to distinguish from EKC. In the adults group, the incidence of corneal involvement was significantly higher than that of other signs, suggesting that the adult patient be treated actively and timely because of the high incidence of corneal lesion, which can cause blurred vision temporarily or permanently. Among the incidence of all typical signs, corneal involvement was the most common accounting for 69.8%, followed by preauricular lymphadenopathy (29.2%), conjunctival edema (17.2%), subconjunctival hemorrhage (16.7%), pseudomembrane (15.1%), and eyelid swelling (15.1%). However, as for the relationship between the serotype of the virus and the clnical manifestation, further research is needed.

The results indicated that the MSI were distributed mostly in the central region of cornea, then in the pericentral region. The central region (0 mm to 3.0 mm diameter) is almost pupillary zone, which is near the visual axis closely linked with the visual quality. The multiple subepithelial corneal infiltrates consist of immune complexes deposited beneath the epithelium in the superficial stroma. They can persist months to years, continuing to impair visual acuity by scattering light, causing irregular astigmatism [25–30]. Because of the impairment of visual function closely correlated with life satisfaction, mental health and daily active ability, more attention should be paid on MSI caused by EKC. That also means, if the disease involved cornea, efficient measure in the treatment should be taken as quickly as possible.

## Conclusions

Appropriate attention should be paid on EKC, because of the considerable proportion of severe cases, the high incidence of corneal lesion, and the high incidence of central region involvement of MSI, which can cause blurred vision temporarily or permanently.

## Data Availability

The datasets used and/or analyzed during the current study are available from the corresponding author on reasonable request.

## Abbreviations

EKC: Epidemic keratoconjunctivitis
MSI: Multiple subepithelial corneal infiltrates
PCR: Polymerase chain reaction
SPK: Superficial punctate keratitis
